# Is costly punishment altruistic? Exploring rejection of unfair offers in the Ultimatum Game in real-world altruists

**DOI:** 10.1038/srep18974

**Published:** 2016-01-07

**Authors:** Kristin M. Brethel-Haurwitz, Sarah A. Stoycos, Elise M. Cardinale, Bryce Huebner, Abigail A. Marsh

**Affiliations:** 1 Department of Psychology, Georgetown University, Washington, DC 20057, USA; 2 Department of Philosophy, Georgetown University, Washington, DC 20057, USA

## Abstract

In the Ultimatum Game (UG), incurring a cost to punish inequity is commonly termed altruistic punishment. This behaviour is thought to benefit others if the defector becomes more equitable in future interactions. However, clear connections between punishment in the UG and altruistic behaviours outside the laboratory are lacking. We tested the altruistic punishment hypothesis in a sample of extraordinarily altruistic adults, predicting that if punishing inequity is predictive of altruism more broadly, extraordinary altruists should punish more frequently. Results showed that punishment was not more prevalent in extraordinary altruists than controls. However, a self-reported altruism measure previously linked to peer evaluations but not behaviour, and on which extraordinary altruists and controls did not differ, did predict punishment. These findings support suggestions that altruistic punishment in the UG is better termed costly punishment and may be motivated by social, but not necessarily prosocial, concerns. Results also support prior suggestions that self-reported altruism may not reliably predict altruistic behaviour.

The term *altruistic punishment* describes the sacrifice of self-interest to punish violations of social norms like fairness or reciprocity^1^,^2^. This behaviour is often modelled using the Ultimatum Game (UG). In the typical UG^3^, an anonymous proposer makes an offer regarding how to split a monetary sum, which the responder may accept or reject. Accepted splits are carried out as proposed. If the split is rejected, both receive nothing. Rejection of any non-zero offer is widely considered irrational^1^,^4^, but players typically reject a proposed monetary offer from another player if it represents less than 20–25% of the total^3^,^4^. In one-shot interactions, such rejections are often described as altruistic because others may benefit from greater equity in future interactions with the punished defector while the player is left objectively worse off^2^,^5^. Further, it is commonly suggested that the purpose of such responses is to encourage fairness and cooperation at the group level^2^,^6^, and that this benefit outweighs the cost to the individual responder^5^.

However, evidence linking costly punishment in the UG to altruism is limited. Rejections of unfair offers in the UG appear to be unrelated or inversely related to prosocial behaviour in other economic paradigms despite the fact that cooperative tendencies across a variety of economic paradigms tend to be correlated, stable over time, and associated with actual helping behaviour^7^,^8^. And unfair offers are usually rejected even in private impunity games in which punishment and the potential for benefiting others have been eliminated^9^,^10^. Consequently, alternate interpretations of costly punishment in the UG have been suggested.

One alternative is that rejection of inequity represents self-interested retaliation, motivated by immediate negative affect^2^,^10^,^11^,^12^,^13^,^14^,^15^,^16^. A second proposal (which is not inconsistent with the first) is that, in keeping with the idea that costly punishment is aimed at punishing violations of social norms like reciprocity, such punishment reflects sensitivity to prosocial group norms. The disposition to punish people who violate group-beneficial norms at a cost yields a reflexive tendency to respond to cooperation with cooperation and to defection with defection, without regard to future payoffs^6^. Such dispositions toward strong reciprocity can sustain high levels of cooperation within groups^17^. Computational models have revealed that strong reciprocity is an evolutionarily stable strategy, and that it supports forms of cooperation that are observed in human populations that cannot be sustained by kin selection or direct or indirect reciprocity^18^,^19^. Strong reciprocity is also consistent with the dual inheritance model of cooperative behaviour suggested by Henrich and colleagues^20^,^21^. According to this model, the adaptive value of strongly reciprocal cooperation, which may be individually costly and without personal benefit, is bolstered by conformist biases that could allow this group-relevant adaptive strategy to be perpetuated through genetic inheritance as well as the social learning of local norms.

The variation in punishment behaviour observed across cultures supports the role of such norms in costly punishment in the UG and other economic paradigms. Some form of costly punishment in the UG is relatively consistent across a wide range of cultures, with extreme inequity being nearly universally punished. But thresholds vary, such that some societies are more punishment-averse, whereas others reject both unfair and hyper-fair offers^22^. This variation is best explained by a tendency toward strong reciprocity, coupled with fairness norms that are dictated by the local economic system, rather than individual-level economic and demographic variables. This suggests that humans may possess a predisposition toward strong reciprocity that supports and sustains cooperation, and that can be modulated by local norms^22^,^23^. Importantly, these processes likely operate at implicit rather than explicit levels in many cases.

We aimed to test the competing hypotheses that rejection of unfair offers in the UG reflects altruism as opposed to normative prosociality. We assessed *non-normative altruism* to dissociate altruism from norm sensitivity. Specifically, we examined UG task performance in a sample of extraordinarily altruistic individuals who had all donated a kidney to a stranger—a behaviour that is simultaneously strongly altruistic and strongly non-normative. Altruistic donors undergo surgery to donate a kidney to an unknown recipient. They receive no compensation and incur various non-monetary costs, including extensive pre-surgical screening and post-surgical pain^24^,^25^, such that altruistic kidney donation satisfies the most stringent definitions of altruism^26^,^27^,^28^. Accordingly, donors typically cite concern for the well-being of the recipient as their top motivation for donating^24^. Altruistic donors typically engage in high levels of other altruistic behaviours, including blood donation and volunteering^24^, consistent with the notion that prosocial tendencies are relatively stable across metrics and across time^7^,^29^,^30^,^31^, and they exhibit patterns of brain structure and activation consistent with heightened socio-emotional sensitivity^32^. But unlike many other forms of prosocial behaviour, altruistic kidney donation is strongly counter-normative, and is often met with scepticism and even derision^24^,^25^.

We assessed *normative altruism* using the Self Report Altruism (SRA) scale^30^. This is a 20-item scale developed to assess self-reports of everyday behaviours such as ceding to others in line and holding doors open. Scores on this scale tend to correspond to other self-reports of prosociality and with peer-reported prosociality^30^. Because public displays of selflessness are an effective means of increasing social status^33^, SRA responses may index prosociality driven by norm conformity^34^, which sustain cooperative interactions but may not be altruistic in nature. Responses gathered using a separate sample of participants support the characterization of the SRA as an index of prescriptively and descriptively normative prosocial behaviours (see *Materials and Methods*). We hypothesised that if costly punishment in the UG stems from altruistic motivations, altruistic kidney donors would engage in increased rejection of inequity relative to controls. Conversely, we hypothesised that if costly punishment in the UG stems from cooperation-sustaining norm conformity^2^, rejection of inequity would correspond more closely to scores on the SRA.

To test these hypotheses, 16 altruistic kidney donors and 28 matched controls played the UG (Table 1). All participants completed preliminary online screening, which inquired about basic demographic information, including age, sex, education, and income; kidney donor status; self-reported normative altruism using the SRA; and self-reported empathy using the Interpersonal Reactivity Index (IRI)^35^. Qualified participants were invited to complete laboratory testing, which included the UG as well as an assessment of IQ and a measure of economic prosociality, the Triple Dominance measure of social value orientation^36^ (see *Materials and Methods*). We included this measure to confirm prior findings that a prosocial social value orientation is not associated with increased costly rejection in the UG^8^,^37^.

The design of the UG (Fig. 1) followed Crockett and colleagues^38^. All participants played the role of the responder in a series of one-shot UGs. In each of the 24 trials, participants decided whether to accept or reject an offer purportedly made by a previous player, which was either fair (45% of the stake), unfair (30%), or very unfair (20%). Offers within each level of fairness were either high ($6) or low ($1), in order to control for responses to monetary value. Before they began the task, participants were informed that their earnings from two trials, selected at random, would be added to their compensation, such that their total payment could be increased by up to $12.

## Results

We first examined how self-reported normative altruism (total SRA score) and social value orientation, as well as self-reported empathy, corresponded to altruistic behaviour by comparing scores on these measures between groups (Table 1). No group differences in normative altruism were observed, *t*(42) = 1.81, *p* = 0.961, nor were group differences in total self-reported empathy, *t*(42) = 0.40, *p* = 0.347. Examining the subscales of the IRI, we identified a group difference in empathic concern, *t*(42) = 2.17, *p* = 0.018, with kidney donors reporting more empathic concern than controls. Kidney donors and controls also did not differ in social value orientation, with similar proportions of both groups characterised as prosocial, χ^2^(1) = 0.97, *p* = 0.325, individualist, χ^2^(1) = 0.92, *p* = 0.337, and proself, χ^2^(1) = 0.54, *p* = 0.463. Examining relationships among these variables, we found that self-reported empathic concern was also correlated with normative altruism, *r*(42) = 0.38, *p* = 0.012, but those with a prosocial social value orientation did not rate themselves more highly on this subscale, *t*(42) = 0.55, *p* = 0.585. Complete comparisons among these assessments can be found in Table 2. Kidney donors and controls did not differ significantly in terms of measured demographic variables with the exception of education level, with relatively more controls having completed a 4-year post-secondary degree, χ^2^(1) = 5.05, *p* = 0.025 (Table 1).

We next assessed patterns of responding in the UG across groups. In keeping with other recent studies of the UG^39^, responses were analysed through the GEE method of logistic regression in which the acceptance or rejection of each offer was the binary response variable. Beginning with a model in which group, fairness, and the interaction between group and fairness were entered as predictors, a main effect of fairness was observed, χ^2^(2) = 72.02, *p* < 0.001; pairwise comparisons confirmed that participants rejected a greater number of very unfair offers than unfair offers, *p* < 0.001, and more unfair offers than fair offers, *p* < 0.001. There was no main effect of group, χ^2^(1) = 0.10, *p* = 0.749, nor a group × fairness interaction, χ^2^(2) = 1.81, *p* = 0.404.

Given the difference in education level between groups, education and the interaction between education and fairness were added to the model. The main effect of education was significant at a trend level, χ^2^(1) = 3.77, *p* = 0.052, such that a higher education level predicted reduced costly rejection overall, *p* = 0.059. There was an interaction between education and fairness, χ^2^(2) = 22.20, *p* < 0.001, in which those with a higher education were less likely to reject very unfair offers, *p* = 0.001 (see Supplementary Fig. S1 online). With education and its interaction with fairness in this model, the predictive value of the group × fairness interaction became significant, χ^2^(2) = 8.69, *p* = 0.013, with kidney donors somewhat less likely than controls to reject very unfair offers; however, pairwise comparisons between kidney donors and controls within each level of fairness remained nonsignificant, all *p* > 0.05 (see Fig. 2).

Next, four alternative models predicting costly punishment were tested, in which group was replaced by normative altruism, self-reported empathy (both total and empathic concern scores), and prosocial social value orientation. Education and its interaction with fairness were dropped from these models because there were no differences in education between the levels of each of these alternate predictors. Self-reported empathy and normative altruism scores were dichotomised via median split. A trend effect of normative altruism, χ^2^(1) = 3.02, *p* = 0.082, was observed, such that those higher in normative altruism were more likely to reject all offers, *p* = 0.075. More importantly, a significant normative altruism × fairness interaction, χ^2^(2) = 13.41, *p* = 0.001, was observed. This effect was supported by pairwise comparisons, such that higher normative altruism scores predicted more rejections of unfair offers, *p* = 0.018 (see Fig. 3).

With regard to total self-reported empathy, neither a main effect, χ^2^(1) = 0.47, *p* = 0.494, nor an interaction with fairness, χ^2^(2) = 3.25, *p* = 0.197, were observed. The empathic concern subscale of the IRI was also examined, since this was the only empathy scale for which a significant difference between kidney donors and controls was observed. While there was no main effect of empathic concern, χ^2^(1) = 0.03, *p* = 0.865, there was an interaction between empathic concern and fairness, χ^2^(2) = 7.58, *p* = 0.023. However, pairwise comparisons between high and low empathic concern within each level of fairness failed to reach significance, all *p* > 0.05. The same pattern was seen with prosocial social value orientation, for which there was no main effect, χ^2^(1) = 1.16, *p* = 0.282, but for which there was a significant interaction with fairness, χ^2^(2) = 11.94, *p* = 0.003. Again, however, pairwise comparisons were nonsignificant, all *p* > 0.05.

Fairness remained a significant predictor in all of the above models, all *p* < 0.001. Significance values for all pairwise comparisons within these models were Bonferroni corrected. Adding group and a group × fairness interaction to the models to control for any effect of group did not change these patterns of results. These patterns of results also remain unchanged when scale variables dichotomized by median split (SRA and IRI) are left continuous in the models described above and then, if a significant scale × fairness interaction is observed, in separate models in which these scale variables predict rejection rates within each level of offer fairness.

### Response latencies

To further examine the rejection behaviour of those scoring high in normative altruism, correlations between rejection rates and reaction times were examined within the unfair offers. Across all participants, a positive correlation was observed at the trend level, *r*(42) = 0.27, *p* = 0.076, such that participants with longer response latencies to unfair offers were more likely to reject unfair offers. Investigating both high and low normative altruism groups separately, we found that this trend was driven by a correlation between response latencies and rejection rates to unfair offers for those high in normative altruism, *r*(20) = 0.47, *p* = 0.029 (see Fig. 4). No comparable correlation emerged between response latency and rejection rate within unfair offers for those low in normative altruism, *r*(20) = 0.01, *p* = 0.980.

### Normative Ratings

Normative ratings of SRA items, costly punishment, and altruistic kidney donation were acquired using a separate online sample (see *Materials and Methods*), which confirmed the descriptive and prescriptive normative nature of SRA behaviours and costly punishment, and the non-normative nature of altruistic kidney donation. With regard to the descriptive frequency of these behaviours, altruistic kidney donation (*M* = 1.65, *SD* = 0.85) is rated as significantly less normative than both costly punishment (*M* = 3.14, *SD* = 0.95), *t*(99) = 11.33, *p* < 0.001, and the average rating for SRA behaviours (*M* = 2.84, *SD* = 0.43), *t*(99) = 13.63, *p* < 0.001 (see Supplementary Fig. S2 online). This is also the case with regard to prescriptive ratings, in which altruistic kidney donation (*M* = 2.94, *SD* = 1.07) is again rated as significantly less normative than both costly punishment (*M* = 3.78, *SD* = 1.24), *t*(99) = 4.75, *p* < 0.001, and the average rating for SRA behaviours (*M* = 3.95, *SD* = 0.71), *t*(99) = 10.06, *p* < 0.001 (see Supplementary Fig. S3 online), despite a significant increase from descriptive to prescriptive ratings for all SRA items, costly punishment, and altruistic kidney donation, all *p* < 0.001. Comparing costly punishment and the average rating for SRA behaviours, costly punishment is descriptively more normative, *t*(99) = 2.64, *p* = 0.005, but the two are prescriptively equivalent, *t*(99) = 0.83, *p* = 0.205. Thus, our characterization of the SRA as a collection of both descriptively and prescriptively normative prosocial behaviours is supported.

## Discussion

Costly rejection of unfair offers in the UG is often characterised as altruistic^5^, despite little direct evidence linking costly punishment to other forms of altruism^7^,^8^. Because inter-individual differences in altruism are moderately heritable and stable^7^,^29^,^30^,^31^, and altruistic kidney donors exhibit altruism across multiple settings^24^, we hypothesised that if costly punishment in the UG reflects simple altruistic motivations, this population would be more likely to punish unfair offers. Our findings did not support this hypothesis, as kidney donors and controls rejected unfair offers at equal rates. Our alternate hypothesis was that if costly punishment instead reflects a reaction to inequity driven by norm-enforcing strong reciprocity^18^,^19^,^20^,^21^,^40^, then costly punishment would more closely correspond to self-reported frequency of normative altruism. Our findings supported this hypothesis, showing a significant relationship between self-reported normative altruism scores and punishment of inequity. These findings are consistent with the idea that punishment of inequity is motivated at least in part by sensitivity to social norms. Both norms of prosociality and norms of punishing defection may result from patterns of behaviour consistent with strategies that are most adaptive or common^20^,^21^, though adopting such patterns of behaviour need not be explicit or conscious. To the extent that norm conformity may drive self-reported, public altruism, this motive may explain both SRA responses and costly rejections of unfair offers in the UG, which are known to follow local cooperative norms^22^,^23^.

This potential role of social norms in SRA behaviours and costly punishment were supported by an online survey in a separate sample. SRA behaviours and costly punishment were rated both as relatively common behaviours within the population, and behaviours that should occur frequently in an ideal world, establishing these behaviours as both descriptively and prescriptively normative. As expected, altruistic kidney donation was rated as significantly less normative, both descriptively and prescriptively. As reviewed by Cialdini and Goldstein^41^, norm conformity may arise from three major motives: accuracy of behaviour with regard to what is correct, affiliation via social approval, and maintenance of a positive self-concept. While each of these may contribute to the tendency to engage in the common, low-cost prosocial behaviours captured by the SRA and also cooperation-sustaining costly punishment, these motives are not compatible with altruistic kidney donation, which is an exceedingly rare behaviour that is not universally viewed as desirable^24^,^25^. That said, we do not assume that norm conformity is necessarily an explicit and proximate motivation for SRA-type behaviours. Instead, the prescriptive nature of such behaviours may result in individuals ultimately seeking to engage in accepted and expected behaviours, although they may not be experiencing these motivations consciously in the moment.

Response latency findings also support these conclusions. Some evidence implicates effortful consideration of cooperative norms and their enforcement in costly punishment^42^^, but see^ 43. Our finding of a positive association between response latencies and rejection rates for unfair offers in those scoring highest in normative altruism is consistent with a “fast to forgive, slow to retaliate” hypothesis^43^,^44^,^45^, in which forced response delays increase rejection rates to moderately unfair offers^44^. In response to such offers, rejection rates for those reporting higher normative altruism in our paradigm may have reflected a stronger motivation to enforce and conform to fairness norms than that experienced by those reporting lower normative altruism. The observation of effects for unfair but not very unfair offers is consistent with previous findings in the UG^38^, and suggests a threshold for rejection, in which those higher in normative altruism may have a higher minimum acceptable offer that falls within the unfair range, but participants do not differ in their tendency to reject very unfair offers. Models in which conflict between choices, rather than an inherently intuitive nature of selfish or prosocial behaviour, predict behaviour^44^,^46^ suggest that longer response latencies would be expected for offers within the UG that are most ambiguous. Unfair offers would fit this criterion, since fair offers are more clearly acceptable and very unfair offers are more clearly unacceptable across respondents.

Retributive motives may be sufficient to motivate costly punishment to unfair offers, in that these offers are usually rejected even in private impunity games in which explicit punishment and the potential for benefiting others have been eliminated^9^,^10^, and when given the opportunity respondents prefer to compensate themselves rather than retaliate against an unfair offer, even when punishment is free^47^. However, punishments increase when they also serve to deter future norm violations^9^, suggesting a distinct role for deterrence in motivating costly punishment. Thus, for those self-reporting greater normative altruism, there may be an added utility of rejecting moderately unfair offers, which satisfies not only a motive to express negative affect but also these individuals’ elevated sensitivity to compliance with cooperative norms.

Highlighting the disconnect between self-reported normative altruism and non-normative altruism, these two forms of altruism were uncorrelated in the present study, with no group difference in normative altruism found for altruistic kidney donors and controls. Donors and controls also did not differ in social value orientation or self-reported total empathy. Separate factors may underlie a lack of group differences for each variable. A prosocial social value orientation is characterised by other-regarding preferences, but is also tied to self-preferences including maximisation of joint outcomes and equality between outcomes. This second facet of a prosocial social value orientation would not be expected to be associated with altruism, which should not be dependent on outcomes for the self^48^. Consistent with this, a prosocial orientation is not the most altruistic of all social value orientations; benefiting another at a cost to one’s own resources would best model altruistic kidney donation, but is not an option in the Triple Dominance measure. Both the IRI and SRA require participants to introspect on how they compare to the average person, potentially making them imperfect proxies for actual altruistic tendencies, which may be orthogonally related to accurate self-perceptions of one’s similarity to population norms. This may be why self-reported empathy and prosociality tend to be reliably associated with one another but not reliably associated with behavioural measures of prosociality^7^,^34^. That kidney donors and controls were similar in terms of these variables is consistent with prior findings that these populations are quite similar on most measures of personality and behaviour except for variables closely related to altruistic behaviour, including neural responses to fearful facial expressions^32^.

Several factors should be considered in situating the results of the present study in the larger context of game theory. It should be noted that our sample size was small compared to many studies of economic games. This is because altruistic kidney donation is an extremely rare phenomenon, with fewer than 1,400 donations recorded in the United States through 2013^49^, rendering recruitment of large samples of donors difficult. Arguably, relationships between UG responses and altruistic behaviour might be identified with a larger sample, although our findings nevertheless make clear that UG responses are more strongly associated with self-reported altruism than actual altruism. It also cannot be determined whether altruistic kidney donors are representative of the “cooperative phenotype” proposed by Peysakhovich and colleagues^7^. Rejections of unfair offers in the UG are also uncorrelated with this “cooperative phenotype,” but whether common mechanisms drive economic cooperation and high-cost altruism is not known. While the UG has been widely used as a measure of costly punishment in general and altruistic punishment in particular, the observed association between normative altruism and costly punishment in the UG should be extended methodologically before conclusions are drawn about norm-enforcing punishment more broadly. In particular, although alternate second- and third-party punishment paradigms are correlated with costly punishment in the UG, that these measures do not group together very strongly suggests divergences that should be further explored with regard to both normative and non-normative altruism^7^. Further research on the interrelationships between laboratory measures of prosociality and real-world altruism may improve our understanding of human altruism.

While the present study cannot determine the exact roles that norm enforcement and self-interested retaliation play in costly punishment, our findings are consistent with a growing body of evidence suggesting that costly punishment in the UG is not predictive of altruism more broadly. Appeals to reputation and norm compliance may be most effective for encouraging cooperation in real-world contexts^50^, and these concerns may also underlie self-reported prosocial acts and costly punishment of defection. But these alternative motives render these cooperative tendencies more self-interested than altruistic – a conclusion supported by our findings in extraordinary altruists. Although punishment in the UG represents a costly behaviour without tangible personal benefit, the intentions of punishers may not reflect simple altruism. These findings add to accumulating evidence that what is often termed altruistic punishment in the UG may be more accurately termed costly punishment.

## Materials and Methods

### Participants

Forty-five healthy adults aged 23 to 56 years (*M* = 43.47, *SD* = 8.51) completed the UG task. The sample included 16 altruistic kidney donors (6 female) and 29 controls (10 female). Sample size was constrained by the extreme rarity of altruistic kidney donors. Donors were recruited from across North America via local and national transplant organisations. All kidney donors’ donations were confirmed via an objective source, such as a letter of confirmation from the transplant centre. Control participants were recruited from the Washington, DC, area via fliers and online advertisements. Data from one control participant were excluded from analyses due to his low response rate (58.34%), 3.9 *SD* below the mean for all participants, leaving 28 controls (10 female). Kidney donors and controls were matched in terms of age, gender, race, household income, and IQ (Table 1). The groups differed in education level, with a greater proportion of controls having attained at least a four-year degree. All participants were free of psychiatric illness, had IQ scores ≥80, and were not currently using any psychotropic medications.

### Procedures

All participants were screened and tested individually. Screening included a preliminary online battery, which assessed basic demographic information and kidney donor status, and included the SRA^30^ and the IRI^35^. For qualified participants, in-person screening followed, including a detailed demographic inventory, a screening inventory for psychiatric illness and medication status, the Triple Dominance measure of social value orientation^36^, the Kaufman Brief Intelligence Test-Second Edition (K-BIT 2)^51^, and the UG.

The SRA measures the tendency to engage in common prosocial behaviours through the endorsement of the frequency of twenty prosocial acts. The IRI^35^ is a multidimensional measure of empathy that provides a total score and four subscale scores: empathic concern, perspective-taking, personal distress, and fantasy. Social value orientation is a measure of social preferences that categorises individuals into three dominant orientations: prosocial, individualist, and competitive^36^. Individualists are characterised by concern with their own gain in a social interaction, regardless of the gain or loss of their interaction partner; competitors are characterised by interest in maximising the disparity between themselves and an interaction partner, in addition to personal gain; and prosocials are characterised by concern for maximising gain for both self and the other, while minimising any disparity between the two, suggesting a particular concern for equity.

Fourteen kidney donors lived more than two hours drive from Washington, DC. For 11 of these participants, travel to Georgetown University and up to two nights lodging were provided through the study. The remaining three kidney donors could not be tested on-site, so the researchers travelled to test them off-site. For the majority of participants tested on-site, testing also included a neuroimaging component^32^; 11 participants participated in the study after the neuroimaging component was completed. Participants were paid $190 for completing the online screening, behavioural testing, and neuroimaging. Participants who did not complete the neuroimaging component were paid $90 for the online screening and testing. This study was approved by the Institutional Review Board at Georgetown University and carried out according to the approved procedures. All participants provided written informed consent prior to study procedures.

### Ultimatum Game

The task was presented using SuperLab Version 4.0 on an Apple desktop computer (or a MacBook laptop computer for participants tested off-site) in a private testing room. The UG was modelled after the procedures of Crockett and colleagues^38^ and was composed of 24 trials, each consisting of a one-shot offer. Participants were instructed that they would be playing a game in which they would play the role of responder, opposite a set of purported past participants, referred to as proposers, who had each submitted a monetary offer to be shared with the participant. To further enhance the credibility of proposers, participants were told before the task began that they would have an opportunity to have their picture taken and make several offers at the conclusion of the task, for use with future participants, though this was only a cover story and the task concluded without participants making their own proposals. The total sum to be split in each trial was referred to as the stake. Proposers’ offers included very unfair offers (20% of the stake), unfair offers (30%), and fair offers (45%). Offers within each level of fairness were either high ($6) or low ($1), in order to control for responses to monetary value. Thus, offer size and fairness were manipulated independently. Very unfair, unfair, and fair offers each made up one third of all trials, evenly split between $1 and $6 offers. Trials were presented in random order, following an initial example trial to confirm participants’ understanding of the task.

**Figure 1:**
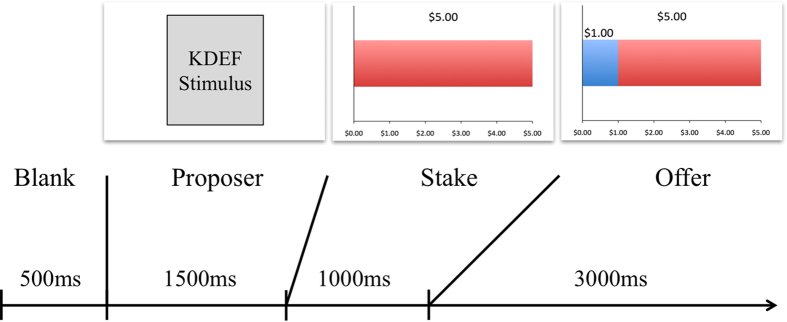
UG task structure. Following a blank screen, participants viewed a picture of the supposed proposer (an image from the Karolinska Directed Emotional Faces picture set, omitted here), the total stake ($5.00 in this case), and the proportion of the stake that was offered ($1.00 in this case). Participants either accepted or rejected the offer during the offer period. This trial structure repeated 24 times, each with a unique proposer, with varying stake and offer sizes.

**Figure 2:**
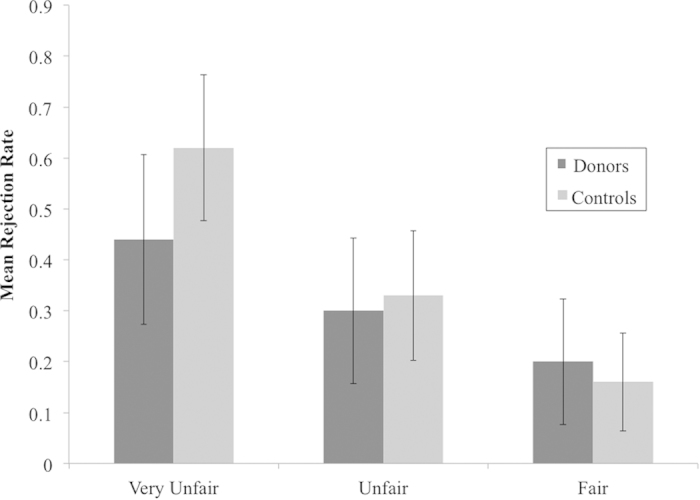
Mean rejection rates broken down by fairness and group. With education and its interaction with fairness in a model with group, fairness, and the group × fairness interaction, the predictive value of the group × fairness interaction was significant, χ^2^(2) = 8.69, *p* = 0.013; however, pairwise comparisons between kidney donors and controls within each level of fairness were nonsignificant, all *p* > 0.05. Error bars represent 95% confidence intervals, based on the SEM.

**Figure 3:**
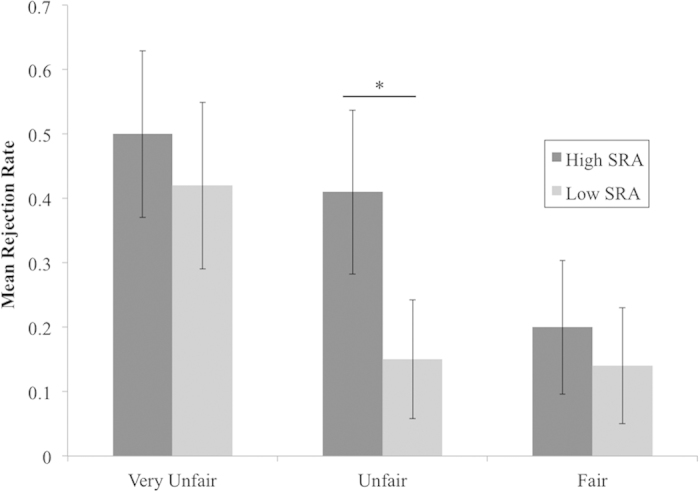
Mean rejection rates broken down by fairness and median split of SRA scores. There was a trend toward a main effect of self-reported altruism, χ^2^(1) = 3.02, *p* = 0.082, in which those with higher self-reported altruism were more likely to reject offers overall, *p* = 0.075. A significant self-reported altruism × fairness interaction, χ^2^(2) = 13.41, *p* = 0.001, was supported by pairwise comparisons, in which those with higher self-reported altruism were more likely to reject unfair offers, *p* = 0.018. *p < 0.05. Error bars represent 95% confidence intervals, based on the SEM.

**Figure 4:**
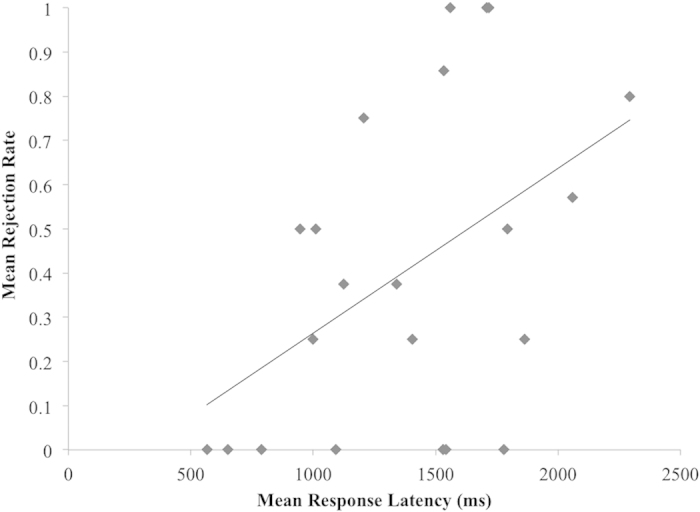
Correlation between rejection rate and response latency for unfair offers in high SRA participants. Longer average response latencies to unfair offers predicted higher rejection rates for participants high in SRA, *r*(20) = 0.47, *p* = 0.029.

In each trial (see Fig. 1), participants first viewed a picture of a proposer, whom participants were told was a previous participant in the study. Proposer pictures were actually 12 male and 12 female Caucasian actors posing neutral expressions from the Karolinska Directed Emotional Faces (KDEF) picture set^52^. Participants were then presented with the stake amount and then the proposer’s offer. As shown in Fig. 1, the division of the stake was illustrated to the participant. Participants could then choose to accept or reject the offer via key press (“1” to accept an offer, “0” to reject it). If the participant accepted, both the proposer and the participant would receive the stated amounts. If the participant rejected the offer, neither party would receive anything. Before they began the task, participants were informed that their earnings from two trials, selected at random, would be added to their compensation, such that their total payment could be increased by up to $12.

### Analysis

Total scores for each participant were calculated for the SRA, and total and subscale scores were calculated for the IRI. Participants were classified into one of the three categories of social value orientation if they selected at least six prosocial, individualistic, or competitive choices out of the nine-item Triple Dominance measure. A proself classification was also computed, based on at least six selections of either individualistic or competitive choices. Group differences in these measures were examined through independent sample t tests and chi-squared tests for independence.

For analysis of the UG, only valid responses made during the offer period of each trial were counted. If participants made multiple responses, only the first valid response was counted. The average response rate across the 24 trials was 92.8% (range: 75–100%). Keeping with other recent studies of the UG^39^, responses were analysed through the generalised estimating equations (GEE) method of logistic regression in SPSS 22. GEE is a semiparametric analysis method that uses generalised linear models while accounting for correlated repeated measurements, thus allowing multiple responses within each condition for each participant. With response to each offer as the binomial response variable, an initial model in which group, fairness, and a group × fairness interaction predicted rejection was examined. Education and its interaction with fairness were then added to this model, due to the group differences in education level between kidney donors and controls. Four alternate models were examined in which group was replaced by self-reported altruism, total empathy on the IRI, empathic concern on the IRI, and prosocial social value orientation. Continuous measures were dichotomised via median split. Fairness was examined as a within-subjects variable while group, education level, and dichotomised self-report measures were examined as between-subjects variables. An exchangeable working correlation matrix was specified, as correlations between repeated trials were expected to be equivalent. A model-based estimator was used for the covariance matrix, since a subject variable was also specified, thus accounting for the repeated nature of within-subject measurements, and also given the relatively small sample size. Finally, correlations between average response latencies and rejection rates, both within unfair offers, were examined as a function of SRA group.

### Online Survey

To confirm our characterization of behaviours listed on the SRA and costly punishment, but not altruistic kidney donation, as normative acts, we conducted an online survey on Qualtrics using Amazon Mechanical Turk. One hundred adult respondents across the United States were presented with each item on the SRA, a description of costly punishment (“Refuse to accept an unfair deal, even if it means both parties get nothing”), and a description of altruistic kidney donation (“Undergo surgery to donate a kidney to a stranger”), and were asked to rate the descriptive and prescriptive frequency of each behaviour. Specifically, on a five-point scale [(1) almost no one, (2) few people, (3) some people, (4) many people, (5) almost everyone] participants rated first what portion of the population ever engages in each behaviour, and then, regardless of real world frequency, what portion of the population should engage in each behaviour, should the opportunity arise, in an ideal world. A free-response manipulation check inquiring how answers were selected for the descriptive and prescriptive sections confirmed that participants were using descriptive and prescriptive frames of reference, respectively. Rating differences were tested with paired sample t tests.

## Additional Information

**How to cite this article**: Brethel-Haurwitz, K. M. *et al.* Is costly punishment altruistic? Exploring rejection of unfair offers in the Ultimatum Game in real-world altruists. *Sci. Rep.* **6**, 18974; doi: 10.1038/srep18974 (2016).

## Supplementary Material

Supplementary Information

## Figures and Tables

**Table 1:** Participant characteristics.

	Kidney Donors (*n* = 16)	Controls (*n* = 28)	*p*
Male/Female (% Male)	10/6 (62.5%)	18/10 (64.3%)	0.906
White/Non-White (% White)	15/1 (93.8%)	24/4 (85.7%)	0.419
Household income			
≥$60,000	11 (68.8%)	17 (63.0%)^a^	0.700
Education			
≥Four-year degree	8 (50.0%)	23 (82.1%)	0.025
Age *M* (*SD*)	44.81 (9.87)	42.71 (7.72)	0.438
IQ *M* (*SD*)	113.19 (12.19)	113.64 (14.19)	0.915
IRI Total	64.81 (13.88)	61.21 (10.93)	0.347
EC	22.94 (4.85)	19.36 (4.52)	0.018
PD	7.31 (4.42)	7.68 (5.58)	0.823
FS	15.31 (5.70)	15.54 (5.47)	0.899
PT	19.25 (3.80)	18.64 (4.14)	0.633
SRA	64.69 (10.01)	64.86 (11.51)	0.961
SVO^b^			
Prosocial	11 (68.8%)	15 (53.6%)	0.325
Individualistic	3 (18.8%)	9 (32.1%)	0.337
Proself	4 (25.0%)	10 (35.7%)	0.463

Note. IRI = Interpersonal Reactivity Index, EC = Empathic Concern, PD = Personal Distress, FS = Fantasy Scale, PT = Perspective-Taking, SRA = Self-Report Altruism Scale, SVO = Social Value Orientation. ^a^One control did not report his/her income. ^b^Six participants (two kidney donors and four controls) were unclassified (not Prosocial, Individualistic, or Competitive) and no participants were classified as Competitive.

**Table 2:** Correlations between donor status, self-reported empathy, self-report altruism, and prosocial social value orientation.

	IRI					SRA	Prosocial SVO	Kidney Donation
	Total	EC	PD	FS	PT			
**IRI**	–	0.69***	0.41**	0.77***	0.58***	0.26	0.15	0.15
**EC**		–	−0.14	0.46***	0.41**	0.38*	0.09	0.36*
**PD**			–	0.12	−0.05	−0.33*	0.10	−0.04
**FS**				–	0.23	0.30*	−0.10	−0.02
**PT**					–	0.33*	0.36*	0.07
**SRA**						–	0.14	−0.01
**Prosocial SVO**							–	0.15
**Kidney Donation**								–

Note. IRI = Interpersonal Reactivity Index, EC = Empathic Concern, PD = Personal Distress, FS = Fantasy Scale, PT = Perspective-Taking, SRA = Self-Report Altruism Scale, SVO = Social Value Orientation. *p < 0.05. **p < 0.01. ***p < 0.05 Bonferroni corrected for 28 comparisons.
