# Supplementary material for: Is costly punishment altruistic? Exploring rejection of unfair offers in the Ultimatum Game in real-world altruists
Source: Sci Rep. 2016 Jan 7;6:18974. doi: 10.1038/srep18974 (PMC4704064; doi:10.1038/srep18974)
Supplement: Supplementary Information [file srep18974-s1.pdf]

Is costly punishment altruistic? Exploring rejection of unfair offers in the Ultimatum Game in real-world altruists

Kristin M. Brethel-Haurwitz[1,*], Sarah A. Stoycos[1,+], Elise M. Cardinale[1], Bryce Huebner[2], and Abigail A. Marsh[1]

[1]Department of Psychology, Georgetown University, Washington, DC 20057, USA.

[2]Department of Philosophy, Georgetown University, Washington, DC 20057, USA.

[*]kb527@georgetown.edu

[+]S.A.S. is now at University of Southern California.

Figure Legends

Figure S1. Mean rejection rates broken down by fairness and education. Given the difference in education level between groups, education and its interaction with fairness were added to the model in which group, fairness, and a group x fairness interaction predicted rejection rates. The main effect of education was marginally significant, $\chi^2(1) = 3.77$, $p = .052$, in which a higher education was associated with less rejection overall, $p = .059$. In an interaction between education and fairness, $\chi^2(2) = 22.20$, $p < .001$, those with a higher education were less likely to reject very unfair offers, $p = .001$. $^{**}p < .01$. Error bars represent 95% confidence intervals, based on the SEM.

Figure S2. Descriptive ratings of altruistic kidney donation, costly punishment, and SRA items. In an a separate online sample, altruistic kidney donation is rated as significantly less descriptively normative than both costly punishment, $t(99) = 11.33$, $p < .001$, and the average rating for SRA behaviours, $t(99) = 13.63$, $p < .001$. Costly punishment is rated as more descriptively normative than SRA behaviours, $t(99) = 2.65$, $p = .005$.

Figure S3. Prescriptive ratings of altruistic kidney donation, costly punishment, and SRA items. In an a separate online sample, altruistic kidney donation is rated as significantly less prescriptively normative than both costly punishment, $t(99) = 4.75$, $p < .001$, and the average rating for SRA behaviours $t(99) = 10.06$, $p < .001$.

Figure S1

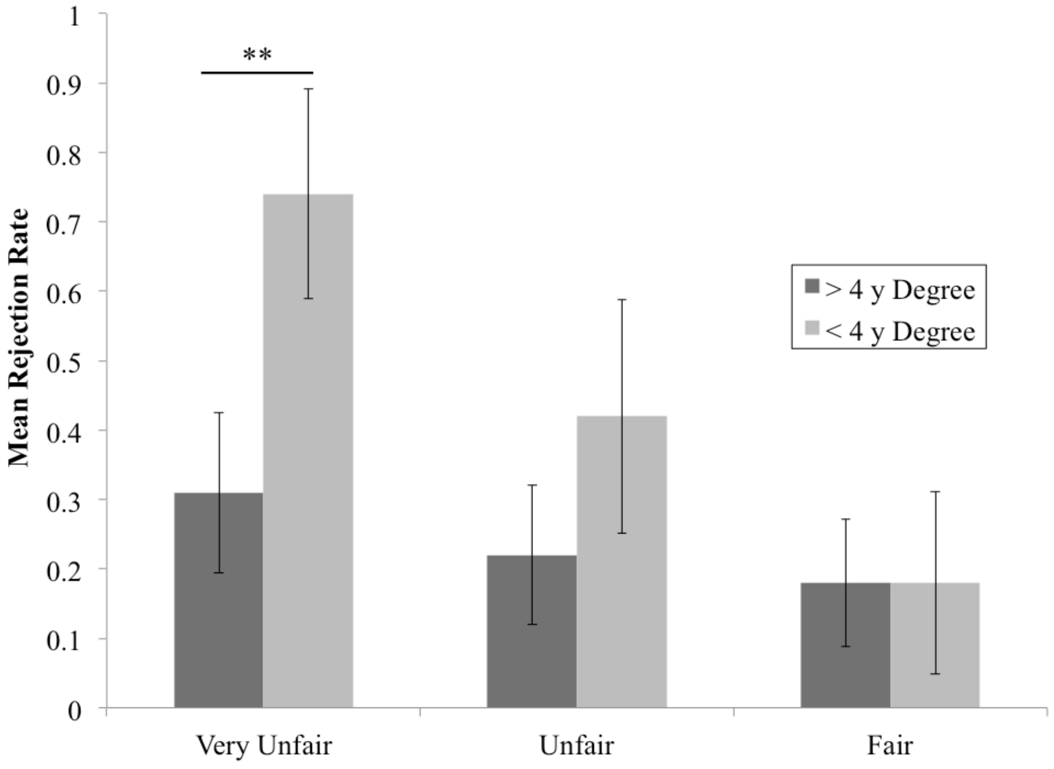

Mean Rejection Rate
1
0.9
0.8
0.7
0.6
0.5
0.4
0.3
0.2
0.1
0
**
> 4 y Degree
< 4 y Degree
Very Unfair
Unfair
Fair

Figure S2

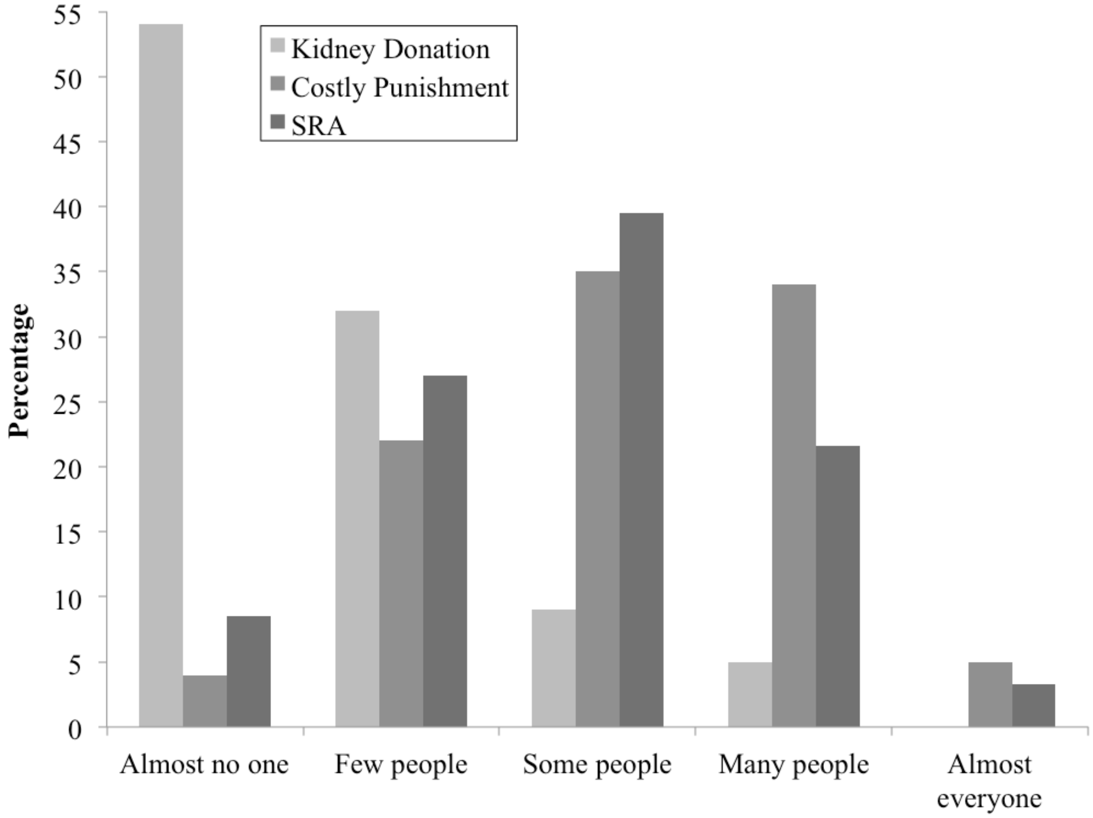
55
50
45
40
35
30
25
20
15
10
5
0
Percentage
Kidney Donation
Costly Punishment
SRA
Almost no one
Few people
Some people
Many people
Almost everyone

Figure S3

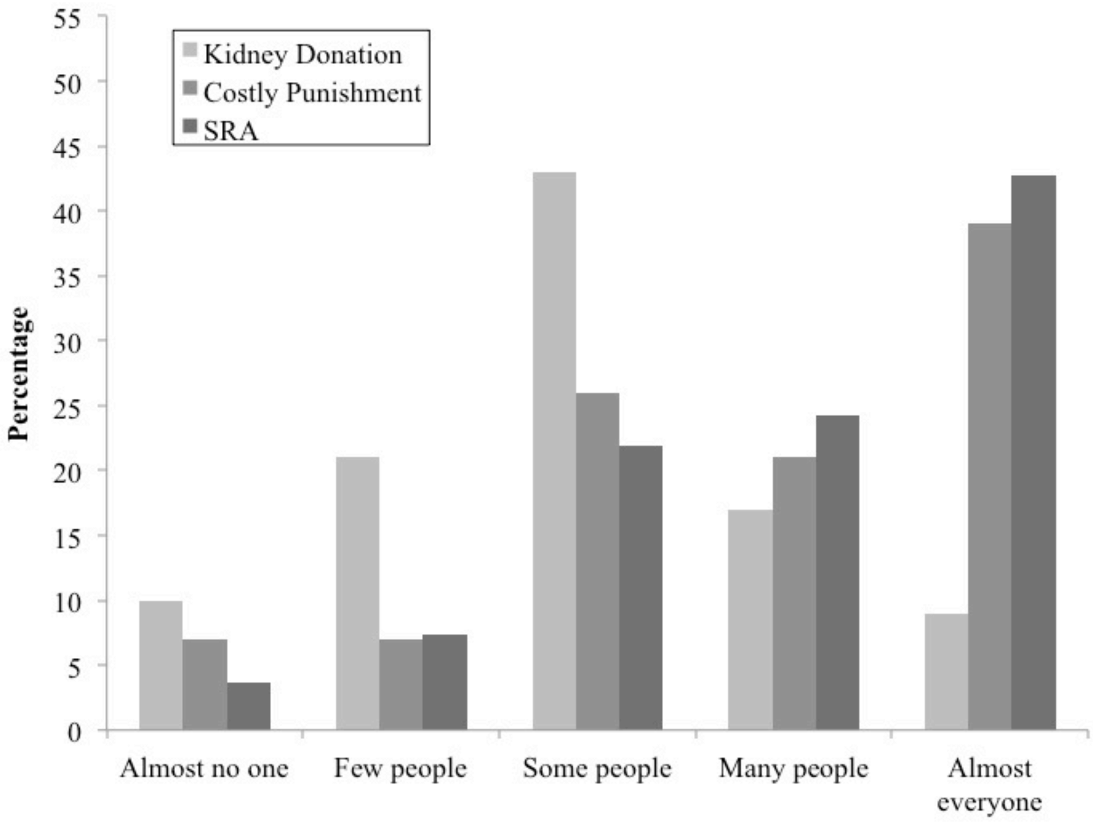
55
50
45
40
35
30
25
20
15
10
5
0
Percentage
Kidney Donation
Costly Punishment
SRA
Almost no one
Few people
Some people
Many people
Almost everyone
